# Outcomes of video training on smoking cessation counseling for nurses

**DOI:** 10.18332/tid/161432

**Published:** 2023-05-05

**Authors:** Surintorn Kalampakorn, Orasa Panpakdee, Rudee Pungbangkadee, Tassanee Rawiworrakul

**Affiliations:** 1 Department of Public Health Nursing, Faculty of Public Health, Mahidol University, Bangkok, Thailand; 2 Nurses Network on Tobacco Control of Thailand, Bangkok, Thailand; 3 Faculty of Nursing, Mahidol University, Bangkok, Thailand

**Keywords:** smoking cessation, nursing education, motivational interviewing, counseling, self-efficacy

## Abstract

**INTRODUCTION:**

Lack of smoking cessation education hinders nurses in providing adequate tobacco cessation counseling. Video training on smoking cessation counseling for nurses was developed and assessed for its short-term outcomes on knowledge and self-efficacy.

**METHODS:**

A quasi-experimental study using pretest–posttest design was conducted with nurses in Thailand in 2020. A total of 126 nurses received online video training. Patient–nurse role-playing was used to demonstrate cessation counseling for smokers who are in the contemplation and preparation stage. Motivational interviewing techniques were emphasized throughout the video. Knowledge and self-efficacy in smoking cessation counseling were assessed pre and post training by a questionnaire.

**RESULTS:**

Comparisons of the pre and post training, mean score of knowledge (10.75 ± 2.39 vs 13.01 ± 2.86, t=7.716, p<0.001) and self-efficacy in smoking cessation counseling (3.70 ± 0.83 vs 4.36 ± 0.58, t=11.187, p<0.001) were significantly increased. These positive learning outcomes were found in nurses with experience and no experience in cessation counseling (p<0.001).

**CONCLUSIONS:**

This study shows that video training can improve nurses’ knowledge and confidence in smoking cessation counseling. It could therefore be included in nursing continuing education to improve nurses’ knowledge and confidence in smoking cessation services.

## INTRODUCTION

Smoking is the leading cause of preventable diseases and death worldwide, with 70% occurring in developing countries^1^. Professional counseling including pharmacological treatment is one of the most effective healthcare interventions to improve smoking cessation rates^2^. Among the healthcare providers, nurses are ideally positioned to advise patients to quit smoking in a wide range of nursing care circumstances. Nurse-led smoking cessation interventions have been effective in quitting smoking in various settings^3^. However, the lack of smoking cessation education hinders nurses from providing adequate tobacco cessation counseling^4^.

A systematic review found that healthcare providers who received specific training had a higher probability of providing smoking cessation intervention to help their patients stop smoking than those who were untrained^2^. Therefore, smoking cessation interventions should be part of standard nursing care and need to be incorporated in nursing training.

The need for flexibility in learning has become increasingly important. Videos are widely used in training as they can give greater flexibility in the teaching and learning process. In addition, video clips promote learning by providing opportunities for preview and review. Previous studies have also demonstrated that the use of video training is a powerful instrument for education and contributes to the enhancement of clinical competencies^5-7^. However, little is known about the effectiveness of video training on smoking cessation counseling. Therefore, this study aimed to assess the outcomes of video training on smoking cessation counseling for nurses and explore whether the outcomes differ between nurses with smoking cessation counseling experience and those with no experience.

## METHODS

The quasi-experimental study using a pretest–posttest design was conducted to explore the outcomes of video training programs with groups of nurses from various healthcare settings in Thailand. Nurses practicing in hospital and primary care settings were invited to participate in the study via e-mail/website/Facebook. The sample size was estimated using the G*power program (software version 3.1)^8^ with a statistical power of 0.95, a significance level of 0.05, and an effect size of 0.4, which gave 84 participants needed. Allowing for attrition, a total of 126 nurses who completed the training and submitted the pre- and post-online assessment were included in this study.

The video training on smoking cessation counseling with patient–nurse role-playing was developed by the researchers with trained and experienced patient actors. Two sessions of role playing, for 10 and 20 minutes, represented two different scenarios, including patients at different stages of change^9^ contemplation and preparation. The goal of the role-play session was to provide participants with guided practice in smoking cessation counseling for patients. The roleplay was based on the 5As counseling approach^10^ where a researcher acted as a smoking cessation provider. Motivational interviewing techniques^11^ were also emphasized as captions throughout the video. The research team developed the scripts provided to the patient actors with content based on clinical experiences and evidence from tobacco control research. Completed video recordings were reviewed by an independent panel of three experts in smoking cessation, health services, and nursing education.

Baseline data were collected using the online questionnaire prior to the 1st session, and the post-intervention data collection occurred immediately after the completion of each video training. The questionnaire collected demographic characteristics and consisted of items assessing knowledge in smoking cessation (10 items for patients at the stage of contemplation and 10 items for patients at stage of preparation), and self-efficacy in smoking cessation counseling (12 items for patients at stage of contemplation and 8 items for patients at stage of preparation). The demographic characteristics assessed were age, gender, education level, work setting, working experience, and cessation counseling experience. Knowledge of smoking cessation was assessed with the responses: Yes (1), No (0), and Do not know (0). Self-efficacy in smoking cessation counseling was assessed using a 5-point Likert scale from certainly not (1), probably not (2), neutral (3), probably (4), and certainly (5). An open-ended question was also provided at the end of the questionnaire for participants to give suggestions they may have on the video. A panel of experts examined content validity. The questionnaire was pretested with 30 nurses who had similar characteristics to the participants. The reliability for knowledge (α=0.764) and self-efficacy (α=0.731) in smoking cessation counseling scales were acceptable.

A descriptive analysis was conducted on participant characteristics. The effectiveness of the intervention on knowledge and self-efficacy in smoking cessation counseling before and after the training was analyzed using paired-sample t-tests.

All participants were informed about the study procedures, voluntary participation, and confidentiality of the responses, and then consent was obtained from each participant through online data collection.

## RESULTS

Among 126 nurses who participated in the study, most were females (88.9%), aged 22–60 years, with a mean age of 41.2 ± 10.5 years. About 60% of participants worked in hospitals, while 40% practiced in primary care settings. Years of work ranged from 1 to 38 years, with a mean of 18.75 ± 10.4 years. Experience in smoking cessation counseling were reported by 60% of the participants. Comparing the characteristics of nurses with experience and those with no experience, no significant differences were found (p>0.05). Therefore, the two groups were considered homogenous. The effectiveness of the video training on knowledge and self-efficacy in smoking cessation counseling is shown in Table 1. There were statistically significant differences in knowledge and self-efficacy between before training and after training (p<0.001). Mean score of knowledge (10.75 ± 2.39 vs 13.01 ± 2.86, t=7.716, p<0.001) and self-efficacy in smoking cessation counseling (3.70 ± 0.83 vs 4.36 ± 0.58, t=11.187, p<0.001) were significantly increased. After the training, significant improvement in knowledge and self-efficacy in cessation counseling for patients at both stages of contemplation and preparation were also found. Concerning the effects of the video training on nurses with experience and no experience in cessation counseling, results show that there were statistically significant differences in knowledge and self-efficacy between before training and after training in both groups (p<0.001), as shown in Table 2. The mean score of knowledge (11.41 ± 2.11 vs 13.01 ± 2.56, t = 4.726, p<0.001) and self-efficacy in smoking cessation counseling (3.87 ± 0.68 vs 4.44 ± 0.49, t =9.757, p<0.001) were significantly increased among nurses with experience in cessation counseling. Also, the mean score of knowledge (9.76 ± 2.45 vs 13.02 ± 3.26, t=6.478, p<0.001) and self-efficacy in smoking cessation counseling (3.47 ± 0.98 vs 4.24 ± 0.69, t=6.725, p<0.001) were significantly increased among nurses who had no experience in cessation counseling.

**Table 1:** Effects of video training on knowledge and self-efficacy in cessation counseling for smokers in contemplation and preparation stage (N=126)

	*Pre-training*	*Post-training*	*t*	*p*
	*Mean ± SD*	*Mean ± SD*		
**Knowledge** (overall)	10.75 ± 2.39	13.01 ± 2.86	-7.716	<0.001
Contemplation	5.68 ± 1.45	6.79 ± 1.51	-7.470	<0.001
Preparation	5.06 ± 1.38	6.22 ± 1.78	-6.38	<0.001
**Self-efficacy** (overall)	3.70 ± 0.83	4.36 ± 0.58	-11.187	<0.001
Contemplation	3.80 ± 0.79	4.41 ± 0.61	-10.82	<0.001
Preparation	3.55 ± 0.94	4.28 ± 0.67	-9.77	<0.001

SD: standard deviation.

**Table 2:** Effects of video training on smoking cessation intervention for nurses with experience (N=75) and no experience (N=51) in cessation counseling

	*Groups*	*Pre-training*	*Post-training*	*Paired t-test*	*p*
		*Mean ± SD*	*Mean ± SD*		
**Knowledge** (overall)	Experience	11.41 ± 2.11	13.01 ± 2.56	-4.726	<0.001
	No experience	9.76 ± 2.45	13.02 ± 3.26	-6.478	<0.001
Contemplation	Experience	5.95 ± 1.43	6.68 ± 1.38	-4.110	<0.001
	No experience	5.29 ± 1.40	6.96 ± 1.68	-6.981	<0.001
Preparation	Experience	5.47 ± 1.11	6.33 ± 1.62	-4.128	<0.001
	No experience	4.47 ± 1.54	6.06 ± 1.99	-4.990	<0.001
**Self-efficacy** (overall)	Experience	3.87 ± 0.68	4.44 ± 0.49	-9.757	<0.001
	No experience	3.47 ± 0.98	4.24 ± 0.69	-6.725	<0.001
Contemplation	Experience	3.95 ± 0.64	4.50 ± 0.54	-9.003	<0.001
	No experience	3.59 ± 0.93	4.29 ± 0.68	-6.629	<0.001
Preparation	Experience	3.74 ± 0.79	4.37 ± 0.58	-7.647	<0.001
	No experience	3.29 ± 1.06	4.16 ± 0.77	-6.363	<0.001

SD: standard deviation.

## DISCUSSION

The current findings show that the video training program enhanced nurses’ ability in cessation counseling. The use of the video can be an effective learning tool since participants can easily remember what is demonstrated in the video clip. Learning through videos is also preferred mainly for flexibility, availability and personalized pace of learning. Participants had the possibility to stop, start, and rewind the video to address their specific needs. Watching the video offers remarkable advantages for increasing and supporting learning, thus contributing to a better understanding of cessation counseling techniques.

The change in knowledge and skills found in this study is similar to that demonstrated by other cessation training^12,13^. This study found that video training, which incorporates a 5As counseling approach and motivational interviewing techniques through role modeling, could enhance knowledge and confidence in cessation practice. This is in line with the participants’ comments on the open-ended question that the video had addressed the content and process of smoking cessation counseling well. Some participants also indicated that they learned about the nurse–patient relationship, thus enabling them to expand and modify their counseling skills according to the individual needs of their future patients. In addition, the training video had positive outcomes not only for those who were new to smoking cessation but also for those who had cessation counseling experience. Therefore, video training can be considered as a useful tool in refreshing and reinforcing smoking cessation skills for nurses. Incorporating video training on smoking cessation counseling into an e-learning platform for continuing nursing education will promote flexibility and accessibility of learning. Also, with further development, the impact of training on improving knowledge can be quickly evaluated using automated tests, thus providing valuable feedback to nurses to improve their learning outcomes in smoking cessation.

### Limitations

Despite the significance of this research, some limitations should be considered. The nature of the pretest–posttest design without a control group and the lack of long-term follow-up limit the impact of the training on the nurses’ behavior and smoking cessation outcome. It is important to note here that the majority of the participants already had some knowledge of smoking cessation, and hence the results may be different from those of other populations.

## CONCLUSIONS

This study shows that video training can improve nurses’ knowledge and confidence in smoking cessation counseling, and it is likely that this training can be useful for continuing education. Although video learning is considered desirable and beneficial for professional development, further investigations are needed on how this training can affect nursing practices and the cessation outcomes of patients.

## Data Availability

The data supporting this research are available from the authors on reasonable request.
